# Supplementary material for: Extracellular Pgk1 interacts neural membrane protein enolase-2 to improve the neurite outgrowth of motor neurons
Source: Commun Biol. 2023 Aug 15;6:849. doi: 10.1038/s42003-023-05223-0 (PMC10427645; doi:10.1038/s42003-023-05223-0)
Supplement: Supplementary file 2 — Supplementary Information [file 42003_2023_5223_MOESM2_ESM.pdf]

Supplementary information

Extracellular Pgk1 Interacts Neural Membrane Protein Enolase-2 to Improve the Neurite Outgrowth of Motor Neurons

Chuan-Yang Fu[1], Hong-Yu Chen[2], Cheng-Yung Lin[3], Shiang-Jiuun Chen[4,5], Jin-Chuan Sheu[6] and Huai-Jen Tsai[1,2,7,*]

[1]Department of Life Science, Fu Jen Catholic University, New Taipei City, Taiwan.

[2] Institute of Molecular and Cellular Biology, National Taiwan University, Taipei, Taiwan.

[3] Institute of Biomedical Sciences, MacKay Medical College, New Taipei City, Taiwan.

[4] Department of Life Science and Institute of Ecology and Evolutionary Biology, National Taiwan University, Taipei, Taiwan.

[5] TechCommon-5, Bioimage Tool, College of Life Science, National Taiwan University, Taipei, Taiwan.

[6]Liver Disease Prevention and Treatment Research Foundation, Taipei, Taiwan.

[7]School of Medicine, College of Medicine, Fu Jen Catholic University, New Taipei, City, Taiwan.

*Corresponding author. Tel: +886 939-919701; E-mails: 012102@mail.fju.edu.tw; hjtsai@ntu.edu.tw

Key Words: Cofilin, Enolase-2, Motor neuron, Pgk1, Zebrafish

Running title: Pgk1 Enolase-2 interaction regulates neurite outgrowth

Contents:

Supplementary Figure 1-18

Supplementary table 1-2

Supplementary Figure 1: Eno1 and Eno2 proteins displayed no transmembrane properties.

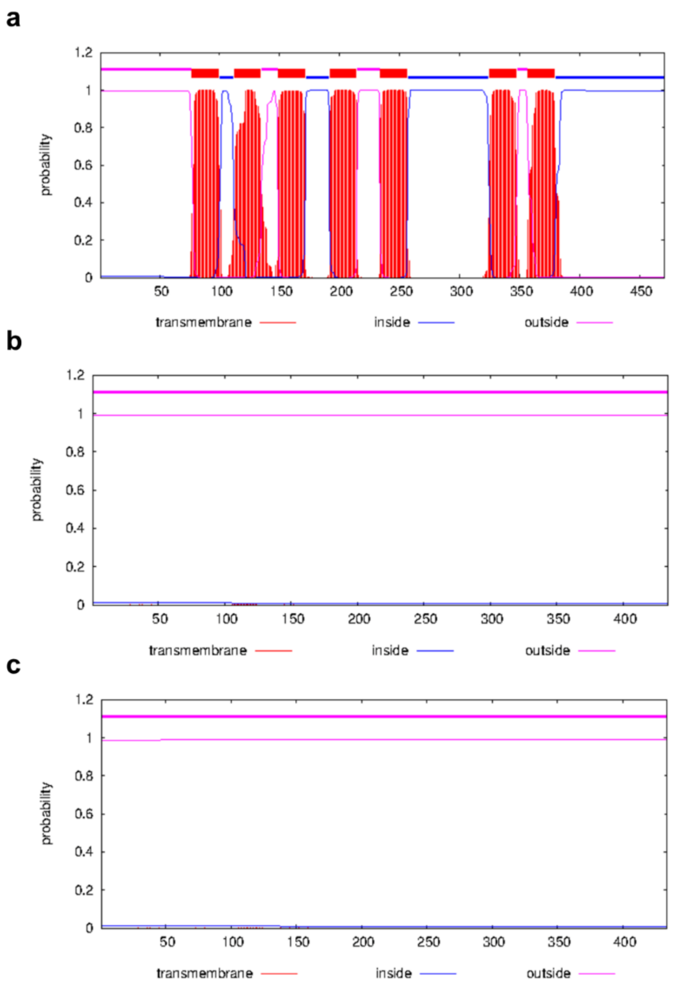

a, b, c Using the TMHMM-2.0 software to analyze the transmembrane properties of proteins: 5-hydroxytryptamine receptor 2A (NP_001233657) **(a)** served as positive control; Eno1 (NP_001020559) **(b)** and Eno2 (NP_001342149) **(c)**. The map is obtained by computing the overall probabilities that the residual bases of amino acids might locate within a helix, inside the membrane and outside the membrane among total sum of all possible paths through the model. Red indicates the transmembrane region, blue indicates inside the membrane, while pink indicates outside the membrane. The horizontal axis is the position of amino acid residues of examined protein.

Supplementary Figure 2: The C-terminal sequences of Eno1 and Eno2 proteins were hydrophilic.

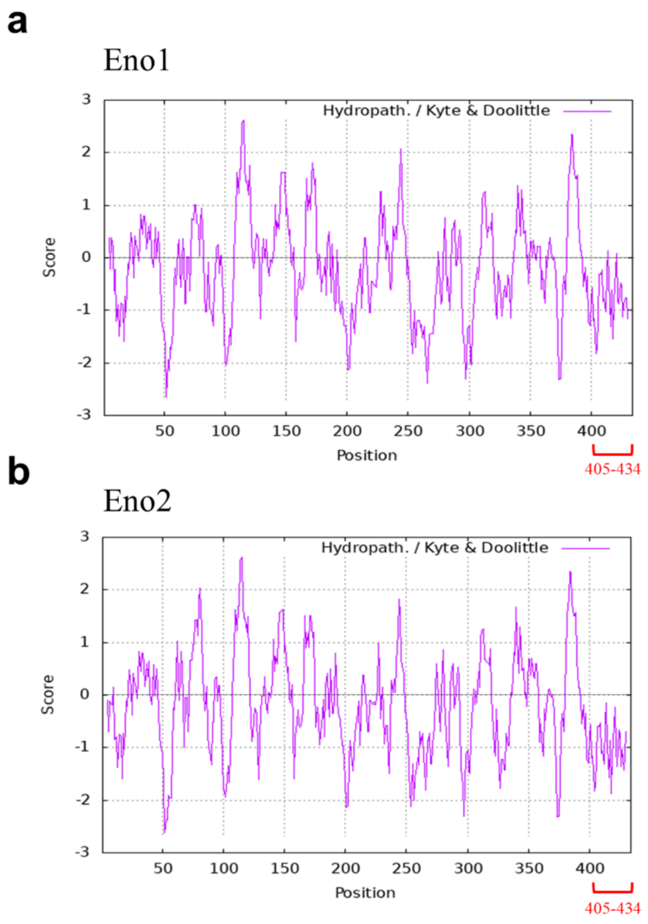

a, b Using the ProtScale-ExPASy software to analyze the hydrophilic structure of **(a)** Eno1 and **(b)** Eno2 proteins. While pink indicates the profile that using the Kyte & Doolittle amino acid scale of ProtScale-ExPASy software to analysis hydrophilic structure. The profile above the midpoint line (Score > 0) indicates the interior region of molecule as hydrophobic, while the profile below the midpoint line (Score < 0) indicates the outside region of molecule as hydrophilic. The horizontal axis is the position of amino acid residues of examined protein. Red brackets indicate the 405th-434th amino acid region located at the C-termini of Eno1 and Eno2.

Supplementary Figure 3: Neurite outgrowth of motor neurons enhanced by reduction of p-Cofilin was induced by the interaction between extracellular Pgk1 and membrane protein Eno2.

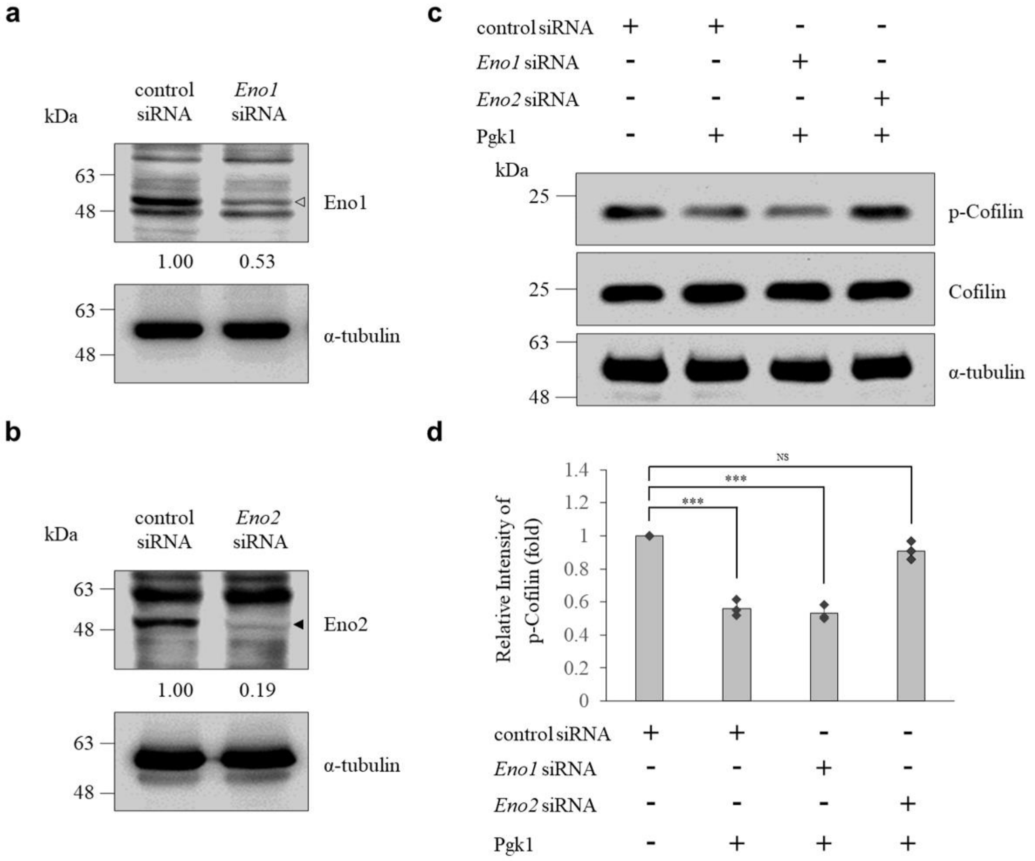

a, b Control and *Eno1*-siRNAs **(a)** and control and *Eno2*-siRNAs **(b)** were individually transfected into neural NSC34 cells. Using antibody against Eno2 to perform Immunoblotting (IB) analysis in order to confirm the effective and specific knockdown by *Eno2*-siRNA, while α-tubulin served as a loading control. The Eno1-positive band shown on Panel **(a)** was marked with an empty arrowhead, while the Eno2-positive band on Panel **(b)** was marked with a solid arrow. **c** Western blot analysis. NSC34 cells were incubated in the presence (+) or absence (-) of mouse Pgk1 and transfected with control siRNA, *Eno1*-siRNA and *Eno2*-siRNA, followed by analyzing the levels of p-Cofilin and

total amount of Cofilin. The α-tubulin served as an internal control. The experiments were performed independently for three times. **d** Statistical analysis. Protein levels relative to each internal control were calculated. The change (in fold) of relative intensity of p-Cofilin against tubulin compared to that of the control group which was set as 1. Data were averaged from three independent experiments and presented as mean ± SD (n = 3). One-way ANOVA, followed by Tukey's multiple comparison test, was used to perform statistical analysis (***, $p<0.001$ and NS, not significant, $p>0.05$). The same protein extracts from each experiment were loaded onto separate Western blots and probed for individual proteins, in parallel.

Supplementary Figure 4: ePgk1 cannot interact with Eno1 on the cell membrane.

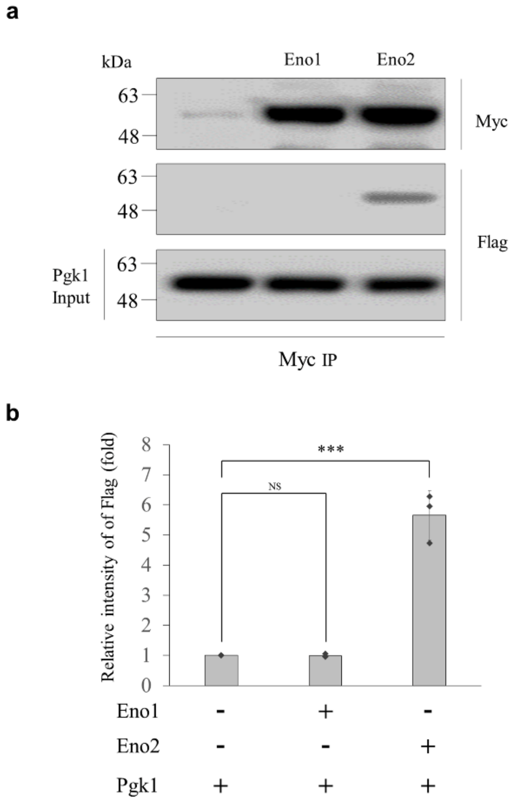

a Cell surface crosslinking-IP. Eno1 and Eno2 fused with Myc were pulled down by Pgk1-Flag. Western blot analysis was used to detect the presence of Pgk1-Flag and Eno2-Myc using antibody against Flag and Myc, respectively. Data were averaged from three independent experiments. **b** Quantification of the intensity of immunoprecipitation shown on cell surface crosslinking-IP. The immunoprecipitation intensity of Eno2-Myc was quantified compared with the intensity of Pgk1-Flag normalized as 1. Data were averaged from three independent experiments and presented as mean ± SD (n = 3). One-way ANOVA, followed by Tukey's multiple comparison test, was used to perform Statistical analysis (***, *p*<0.001 and NS, not significant, *p*>0.05). The same protein extracts from each experiment were loaded onto separate Western blots and probed for individual proteins, in parallel.

Supplementary Figure 5: The effect of incubation of extracellular Pgk1 and injection of *eno2*-MO on the neurite outgrowth of motor neurons in zebrafish embryos.

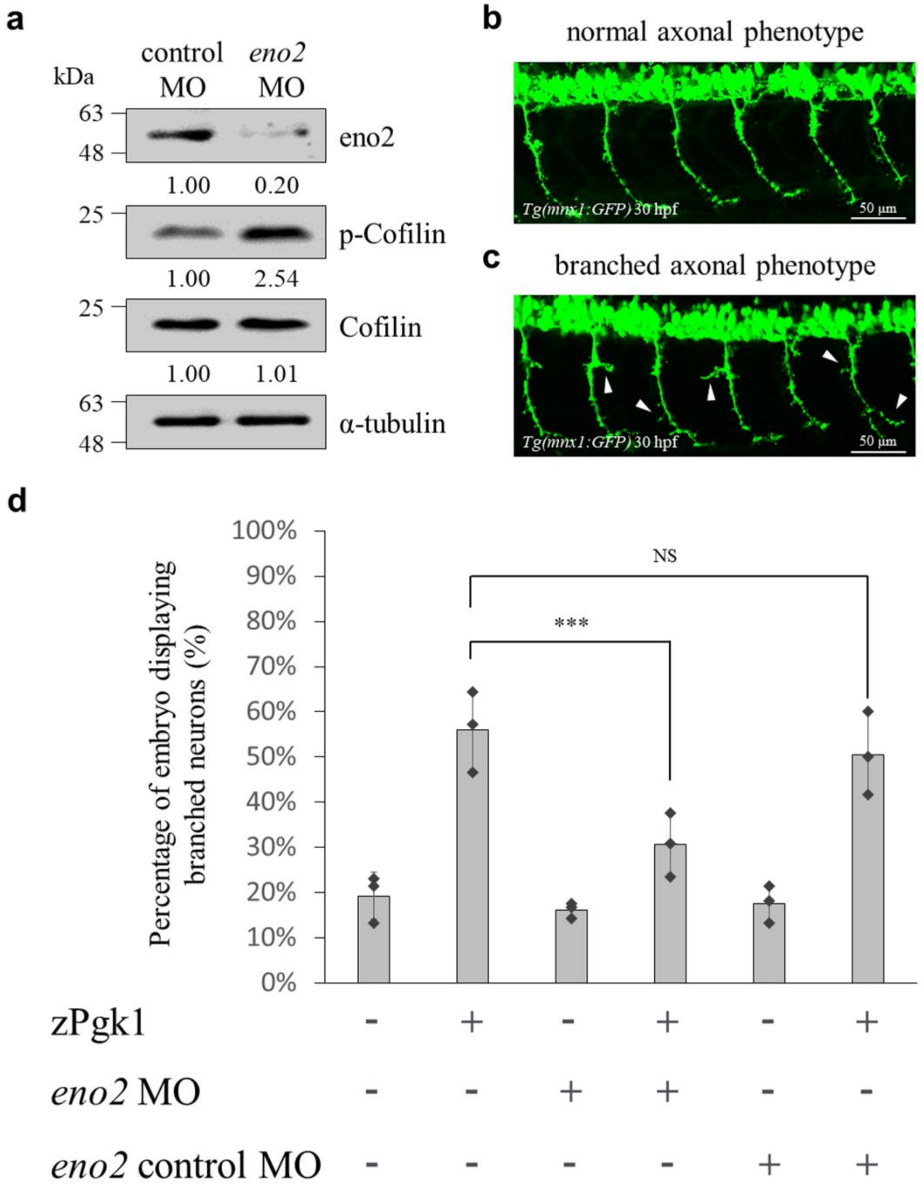

a control-MO and *eno2*-MO were individually injected into one-celled embryos. Antibody against Eno2 was used to perform Immunoblotting analysis with the aim of confirming the effective and specific knockdown by *eno2*-MO. The relative levels of Eno2, phosphorylated Cofilin at S3 (p-Cofilin) and total Cofilin were quantified based on the values compared to those of control MO set as 1 and shown on each lane below. The α-tubulin served as an internal control **b, c** Two phenotypes exhibiting the branched motor neurons shown on embryos from zebrafish transgenic line *Tg(mnx1:GFP)* were observed at 30 hpf under fluorescence microscopy: normal axonal phenotype neurons **(b)** and branched axonal phenotype neurons (white and arrows) **(c)**. Scale bar: 50 μm. **d** Statistical analysis. The percentage of embryos having branched motor neurons among total examined embryos treated in the presence (+) or absence (-) of zPgk1 incubation for 24 hr and *eno2*-MO injection. The injection of control-MO served as control. The experiments were performed independently for three times. Data were averaged from three independent experiments and presented as mean ± SD (n = 3). One-way ANOVA, followed by Tukey's multiple comparison test, was used to perform statistical analysis (***, $p<0.001$ and NS, not significant, $p>0.05$). The same protein extracts from each experiment were loaded onto separate Western blots and probed for individual proteins, in parallel.

Supplementary Figure 6: Binding between Pgk1 and Eno2 was impeded in the presence of Eno2-specific antibody.

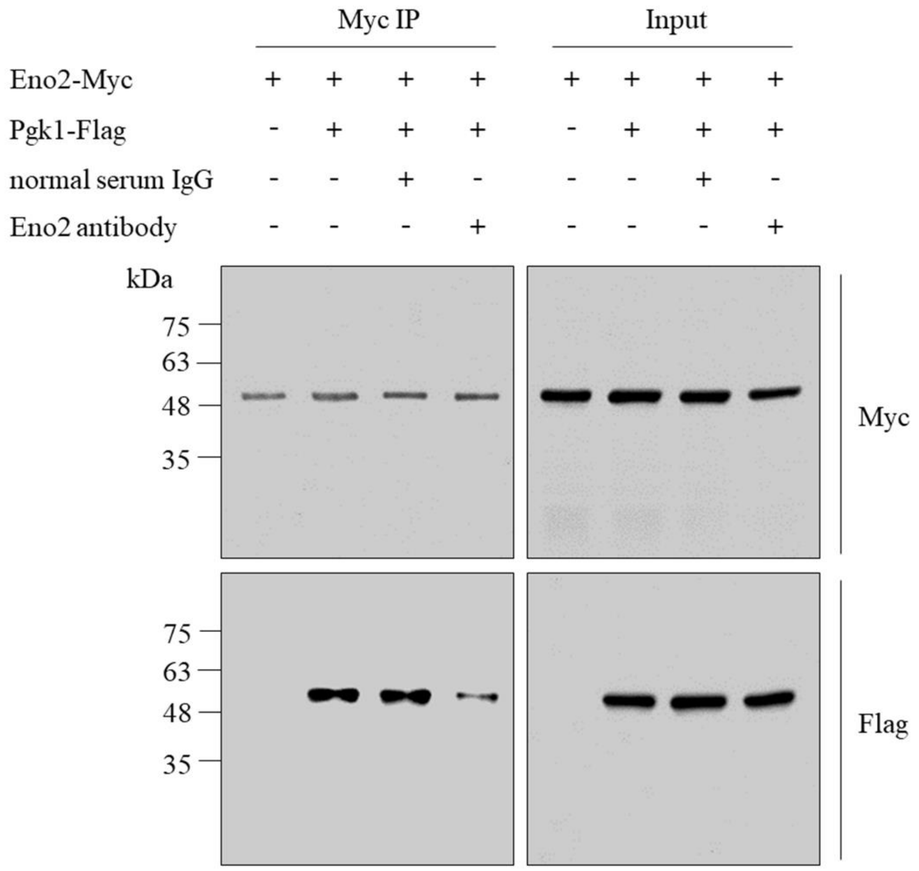

Eno2-Myc-expressing Sf21insect cells were incubated in the medium containing Pgk1-Flag, followed by cell surface crosslinking-IP using anti-Myc (IP: Myc) and then assessed by Western immunoblot (IB) using either anti-Flag (Flag) or anti-Myc (Myc). Eno2-specific antibody was used to perform the assay whereby interaction was blocked, while normal serum immunoglobulin (IgG) served as negative control. Input represented 10% of total cell extracts used for each immunoprecipitation. The experiments were performed independently for three times. The same protein extracts from each experiment were loaded onto separate Western blots and probed for individual proteins, in parallel.

Supplementary Figure 7: Extracellular Pgk1 did not decrease the level of regulatory proteins involved in the p-p38/p-Limk1/p-Cofilin pathway in Eno2-knockdown NSC34 cells.

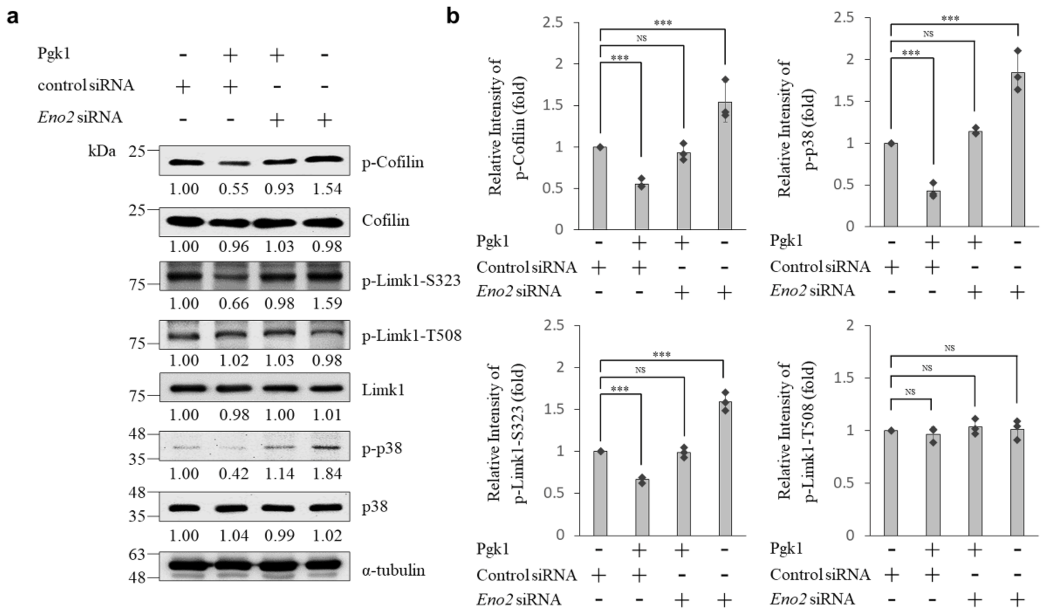

a Western blot analysis. NSC34 cells were incubated in the presence (+) or absence (-) of Pgk1 combined with either control siRNA or *Eno2*-siRNA, followed by analyzing the levels of regulatory proteins, as indicated. The α-tubulin served as an internal control. Protein levels relative to each internal control set as 1 were presented at each lane. The experiments were performed independently for three times. **b** Quantitative and statistical analyses. Data were averaged from three independent experiments and presented as mean ± SD (n = 3). One-way ANOVA, followed by Tukey's multiple comparison test, was used to perform statistical analysis (***, $p<0.001$ and NS, not significant, $p>0.05$). The same protein extracts from each experiment were loaded onto separate Western blots and probed for individual proteins, in parallel.

Supplementary Figure 8: Unedited/uncropped western blot gels for Figure 1

Fig. 1b

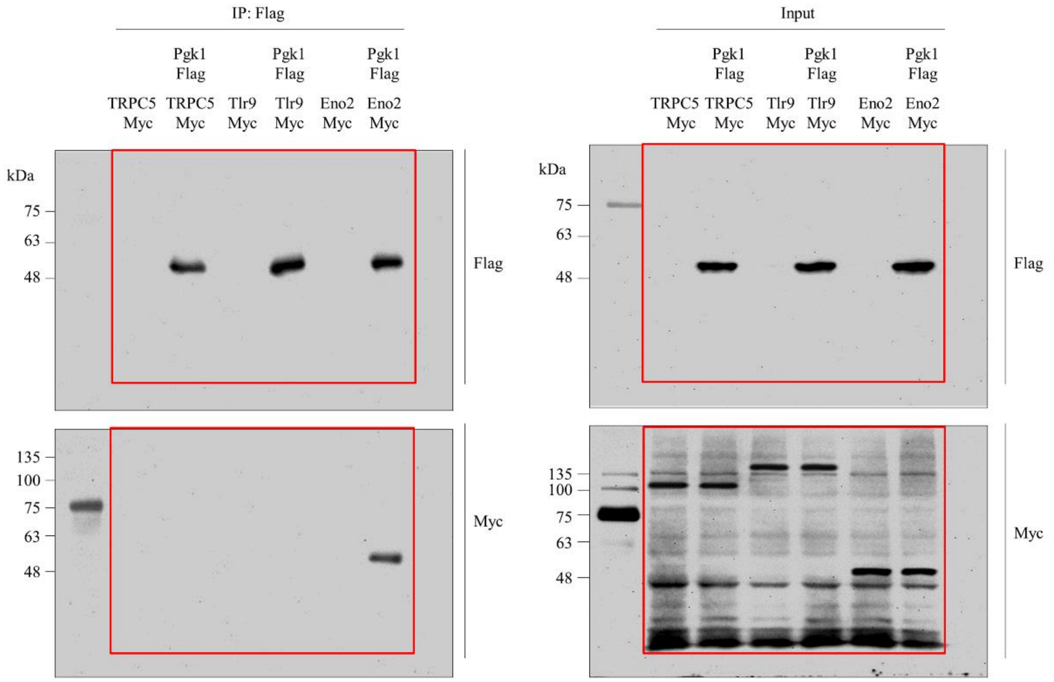

Fig. 1c

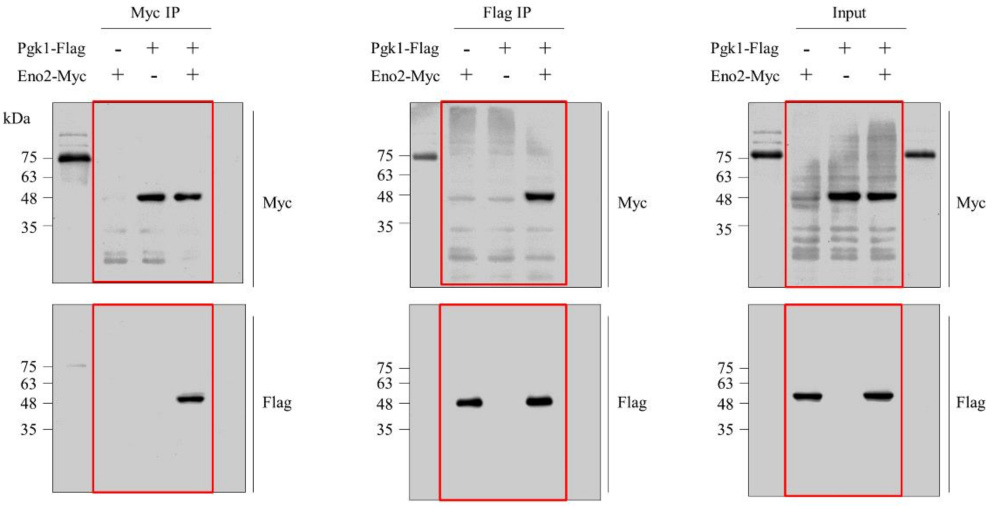

Supplementary Figure 9: Unedited/uncropped western blot gels for Figure 3

Fig. 3b

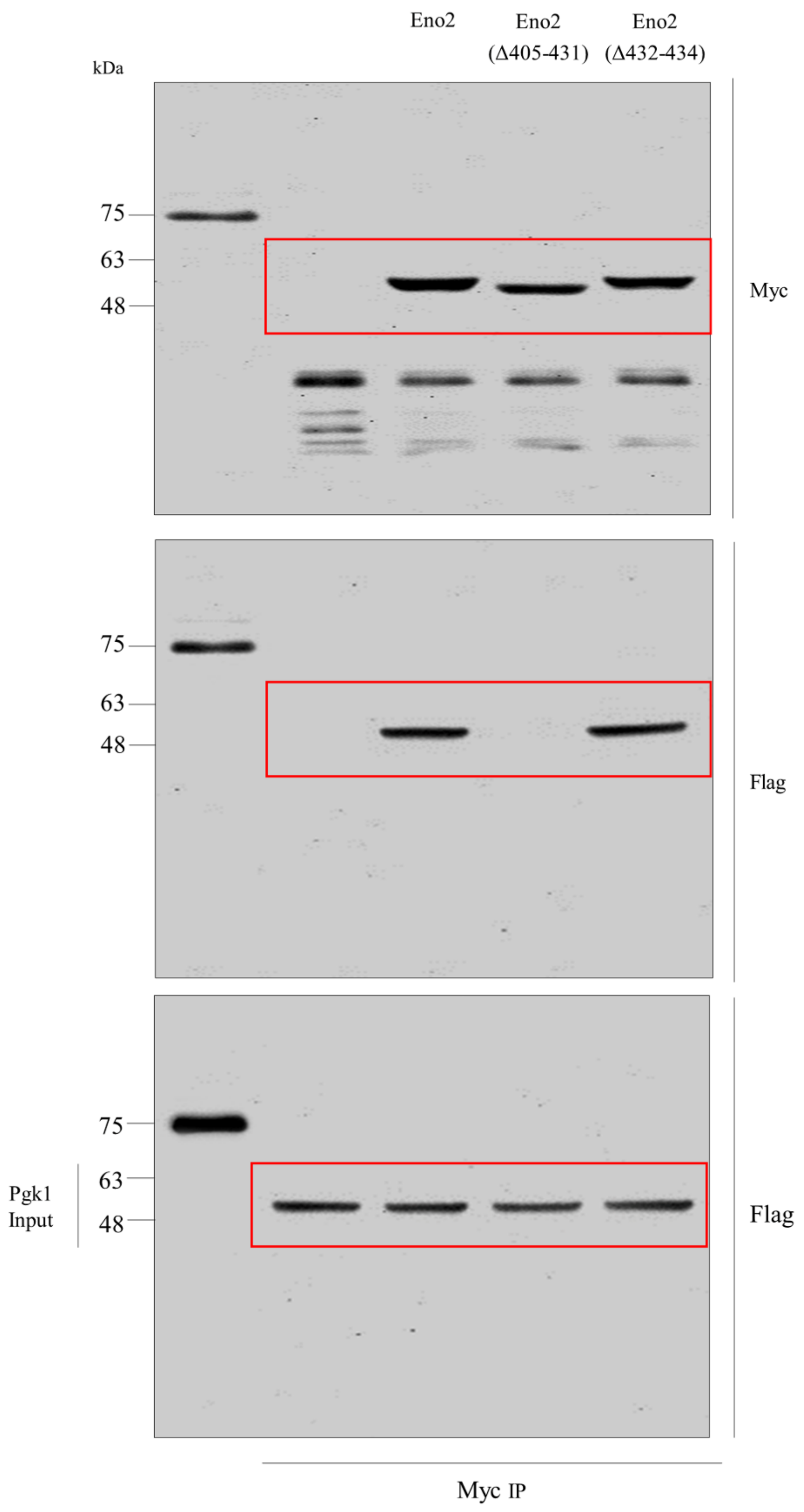

Supplementary Figure 10: Unedited/uncropped western blot gels for Figure 4

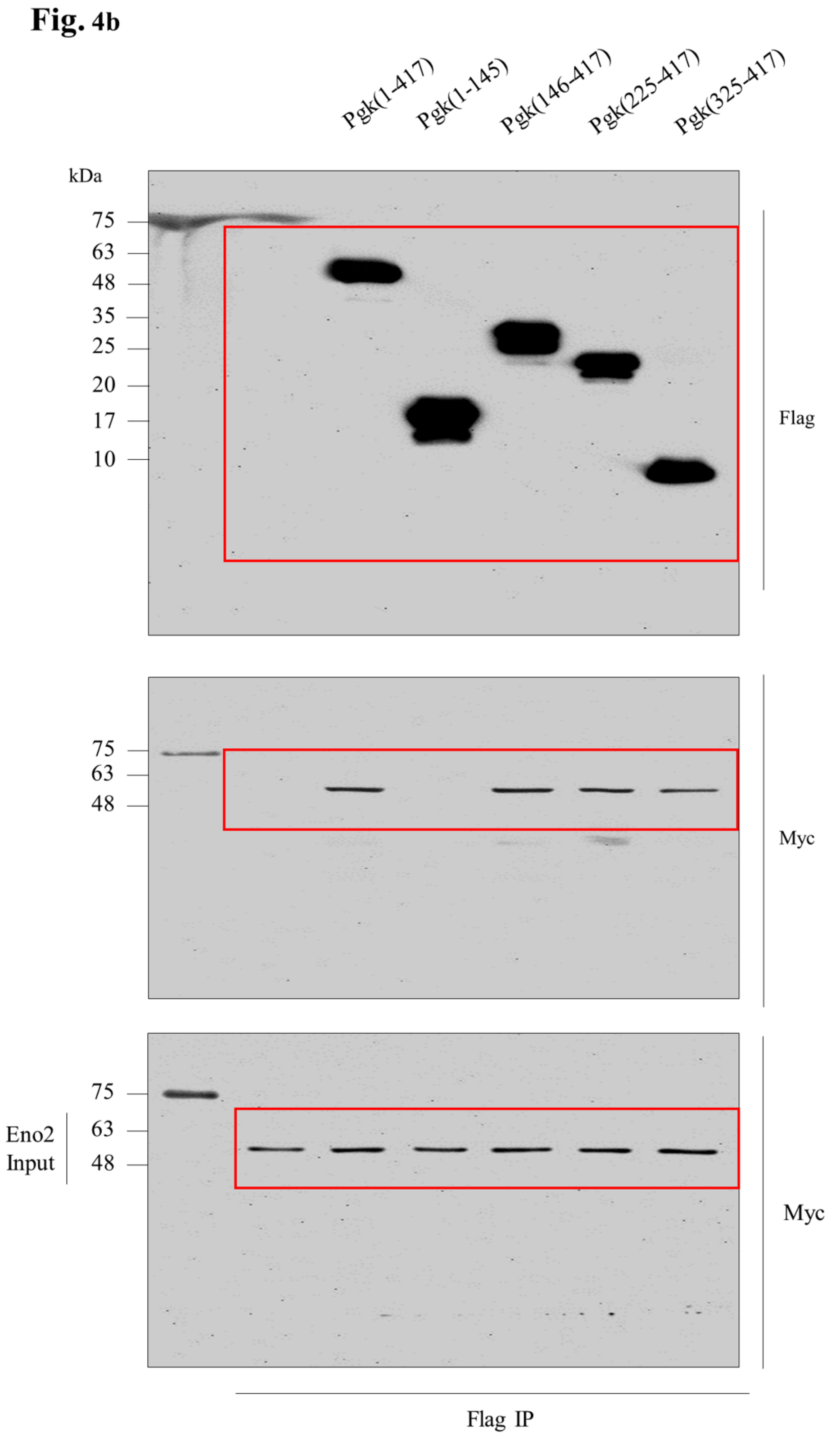

Supplementary Figure 11: Unedited/uncropped western blot gels for Figure 5

Fig. 5e

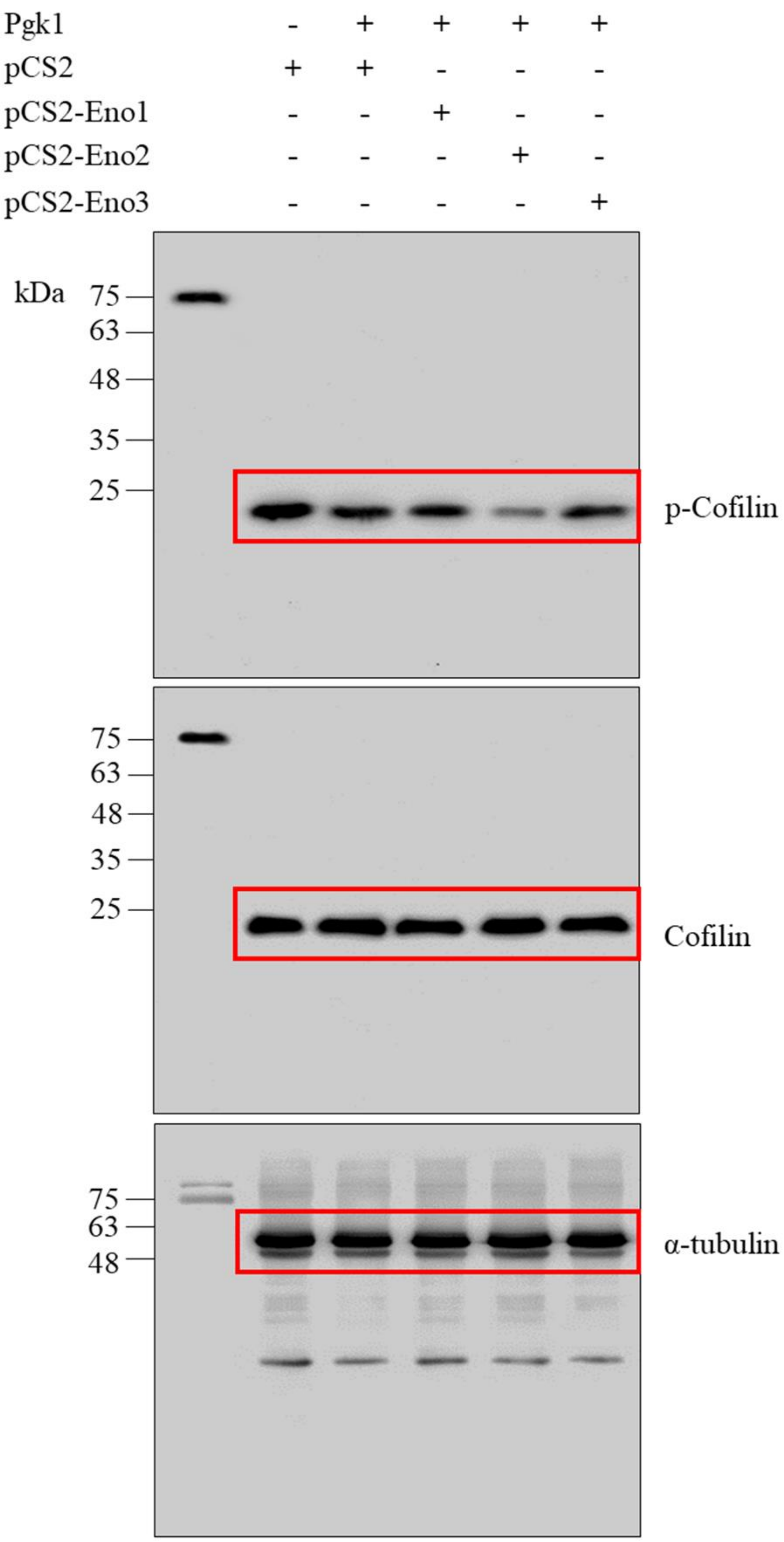

Supplementary Figure 12: Unedited/uncropped western blot gels for Figure 6

Fig. 6d

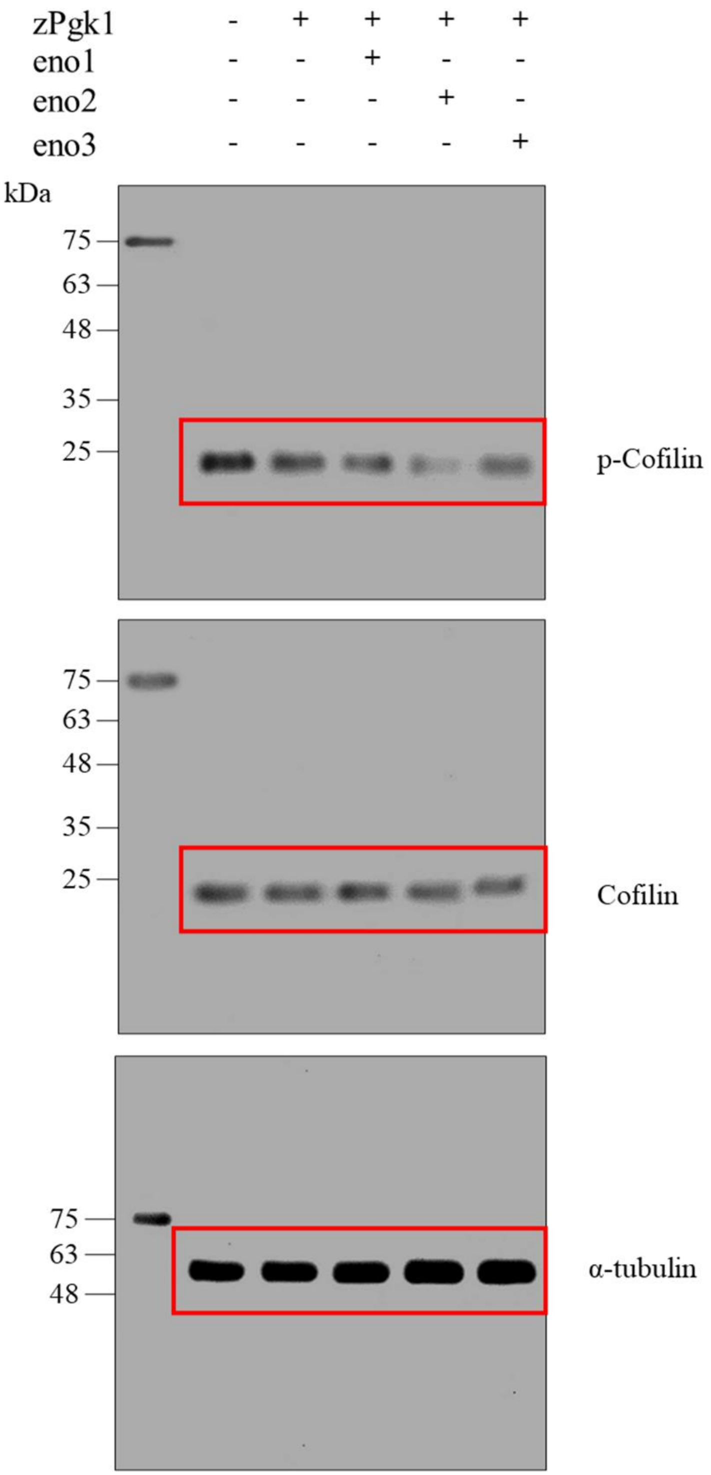

Supplementary Figure 13: Unedited/uncropped western blot gels for Figure 7

Fig. 7a

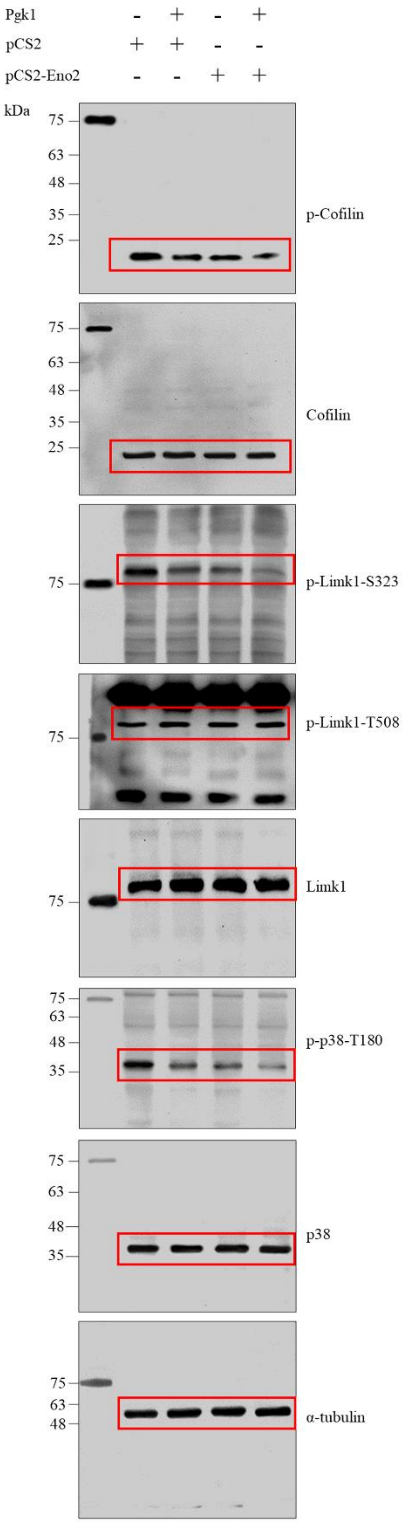

Fig. 7c

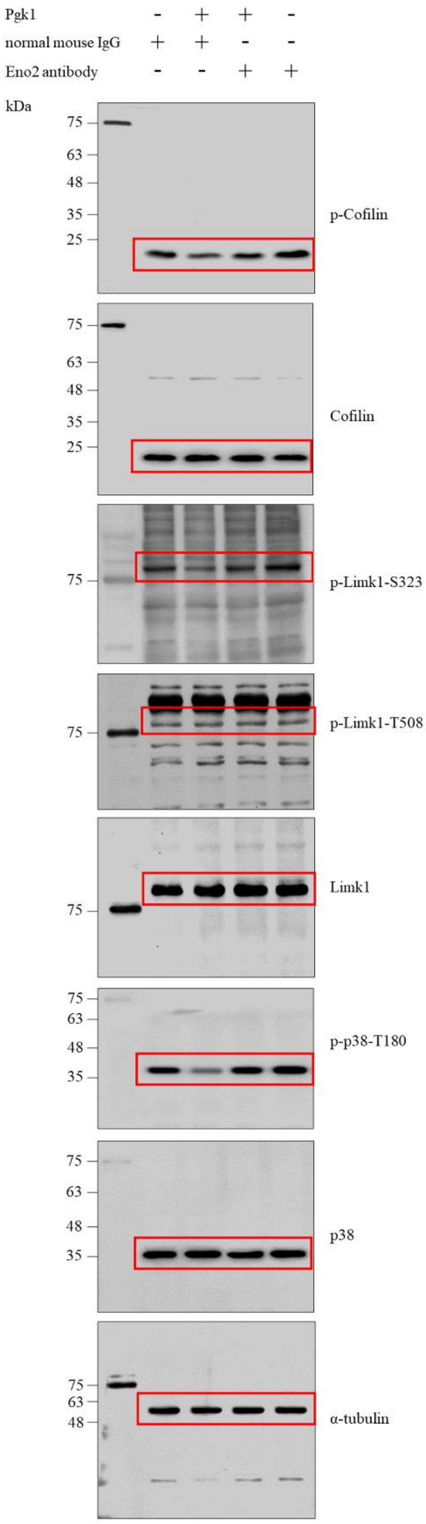

Supplementary Figure 14: Unedited/uncropped western blot gels for Supplementary Figure 3

Supplementary Fig. 3a

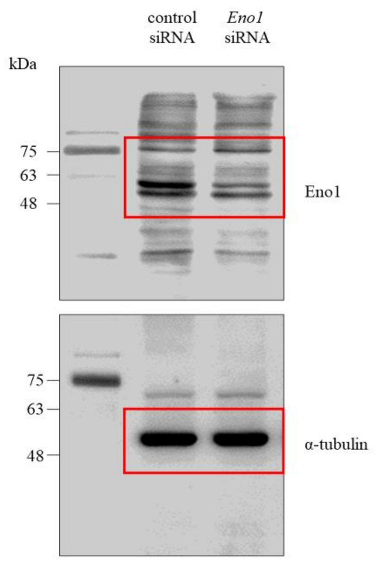

Supplementary Fig. 3b

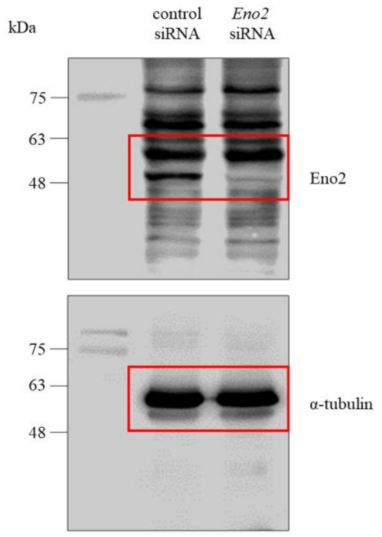

Supplementary Fig. 3c

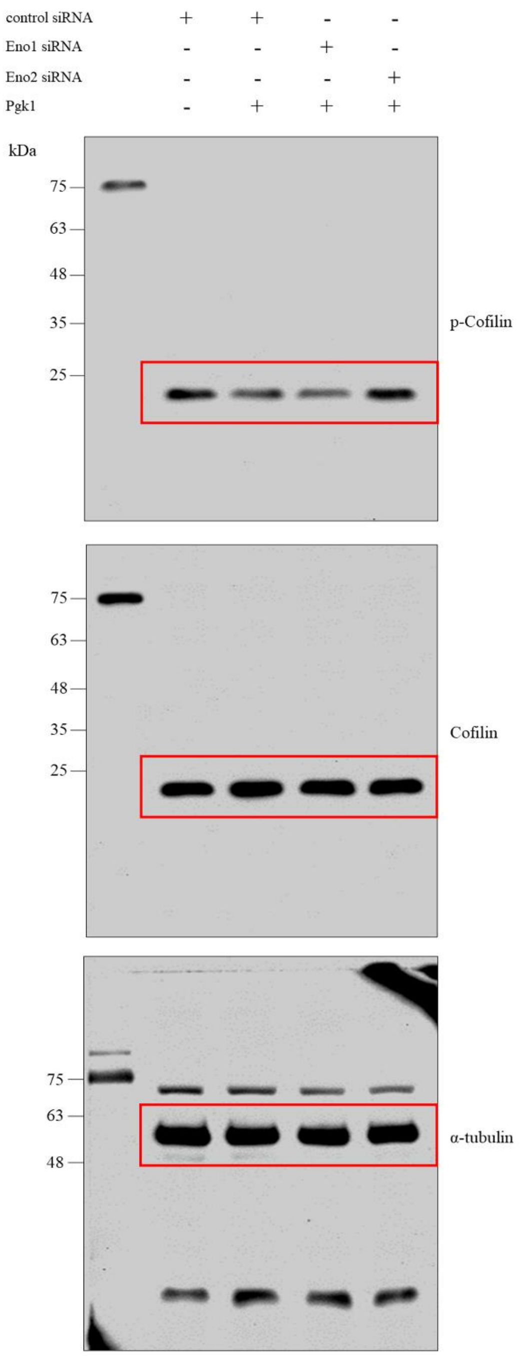

Supplementary Figure 15: Unedited/uncropped western blot gels for Supplementary Figure 4

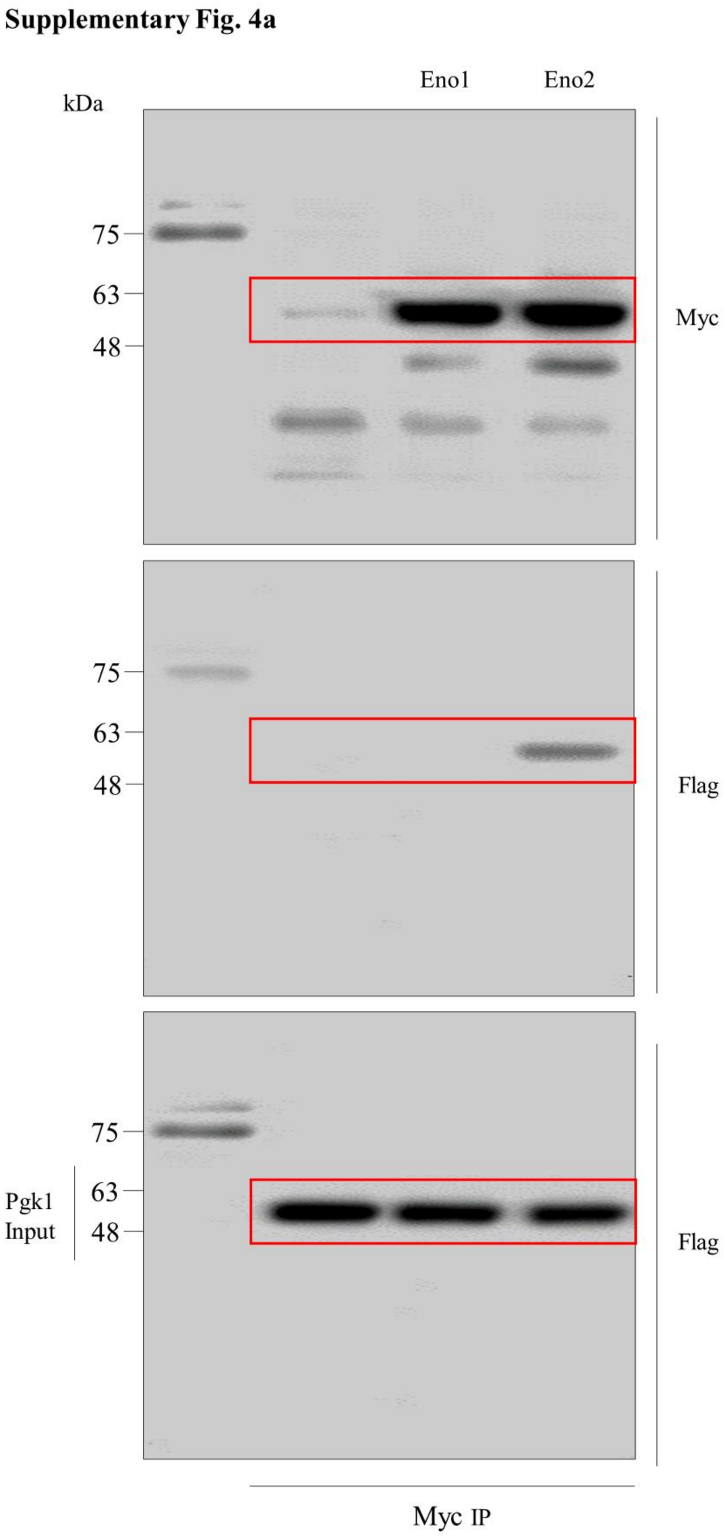

Supplementary Figure 16: Unedited/uncropped western blot gels for Supplementary Figure 5

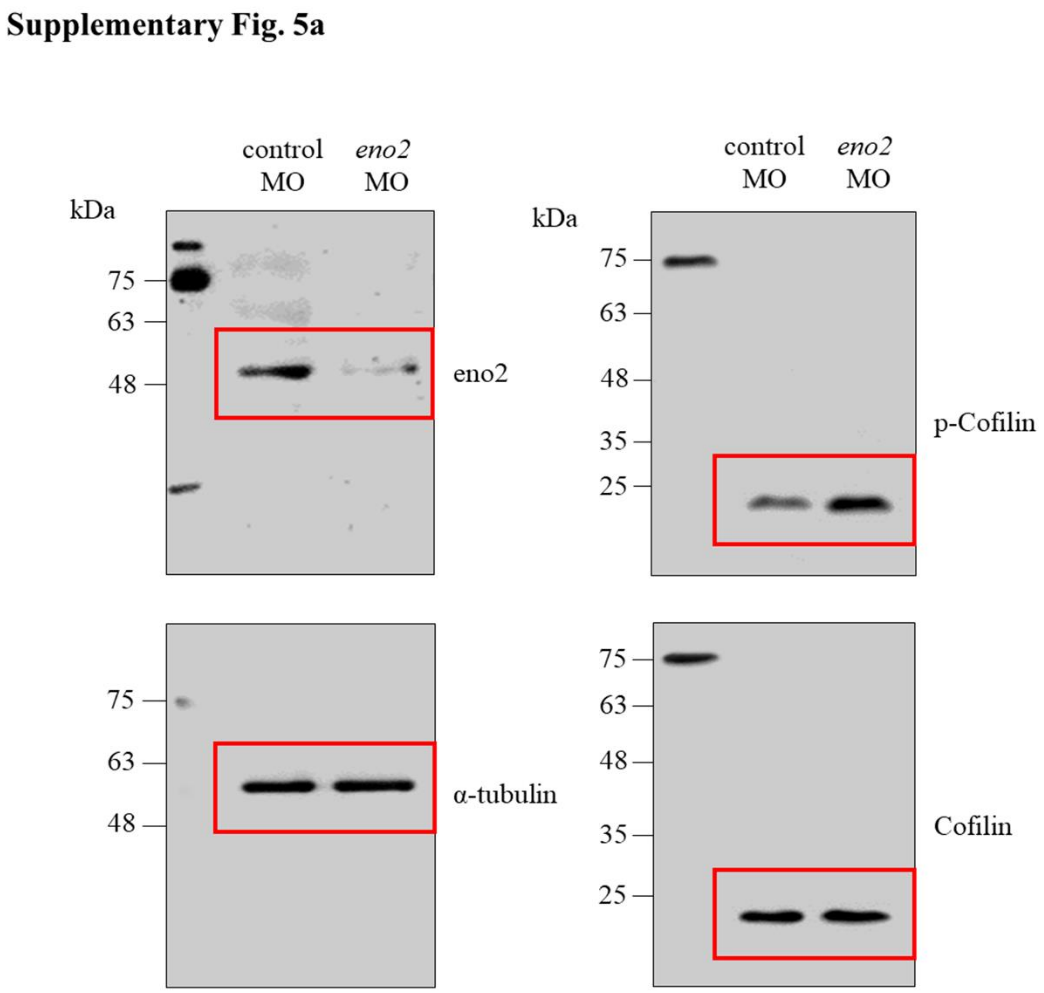

Supplementary Figure 17: Unedited/uncropped western blot gels for Supplementary Figure 6

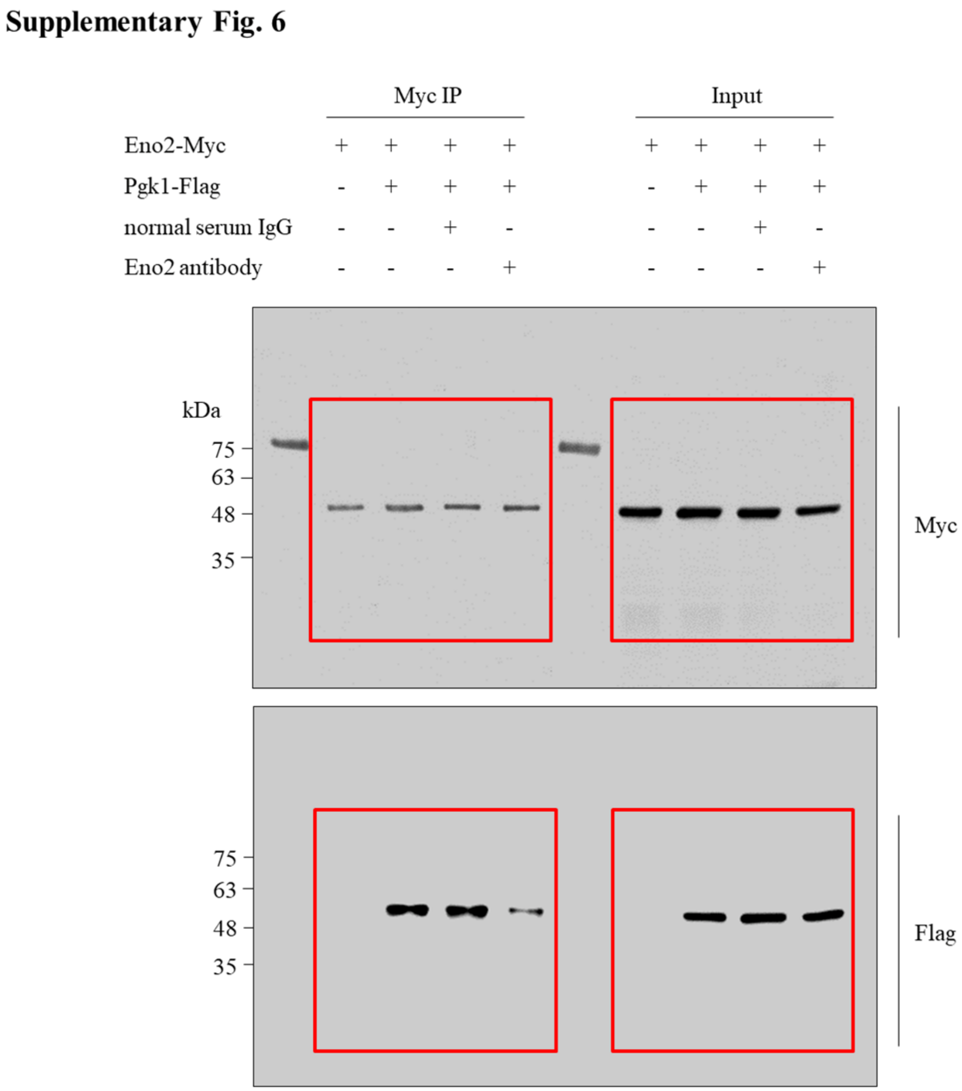

Supplementary Figure 18: Unedited/uncropped western blot gels for Supplementary Figure 7

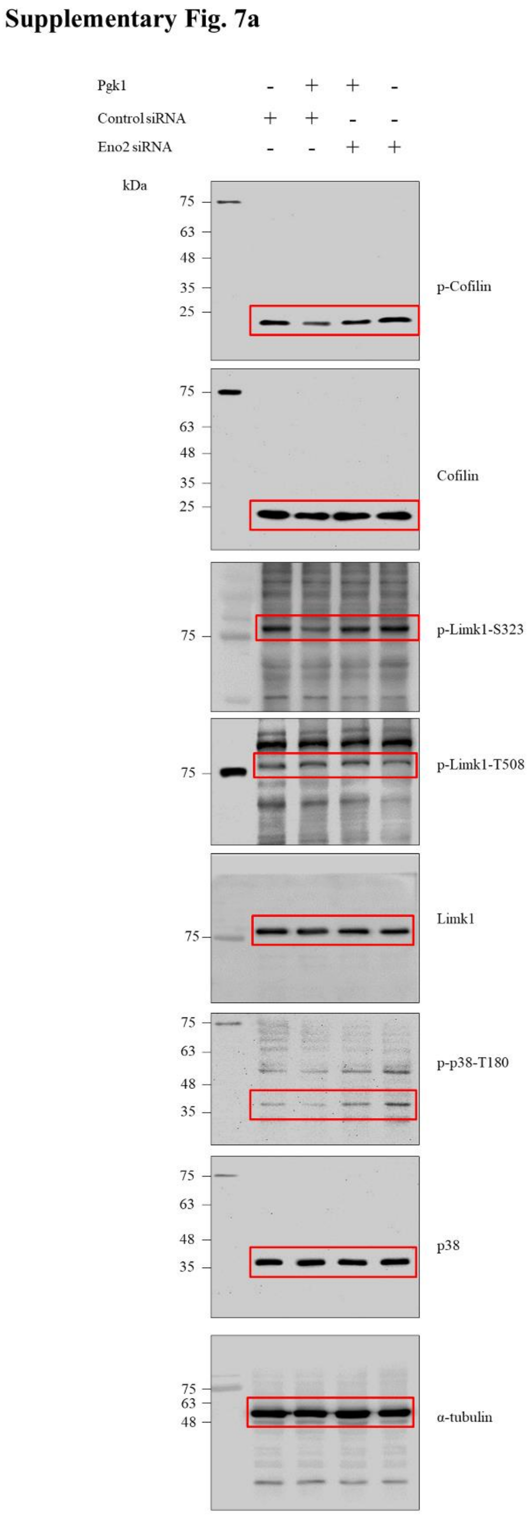

Supplementary Table 1. A profile of putative genes and their encoded proteins obtained by LC-MS/MS analysis after the Pgk1-Flag fusion protein was used to pull down the membrane proteins extracted from NSC34 cells.

Gene ID	Protein name	Proteins abbreviated nomenclature	Numbers of peptides detected	Scores
NM_146153	Thyroid hormone receptor-associated protein 3	THRAP3	1	18
NM_001159321	Glutamate receptor, ionotropic, delta 2 interacting protein 1	Grid2ip	2	35
NM_008166	Glutamate receptor, ionotropic, delta 1	Grid1	3	68
NM_031178	Toll-like receptor 9	Tlr9	5	122
NM_009428	Short transient receptor potential channel 5	TRPC5	5	113
NM_001293685	Vesicle transport through interaction with t-SNAREs 1A	Vti1a	3	53
NM_001177371	Drebrin 1	Dbn1	2	39
NM_001355220	Enolase 2	Eno2	5	118
NM_001081124	MAP7 domain containing 2	Map7d2	2	33
NM_173862	Family with sequence similarity 83, member A	Fam83a	1	25
NM_177376	Myosin IIIB	Myo3b	2	39
NM_054053	adhesion G protein-coupled receptor V1	GPR98	3	98
NP_033412	Tight junction protein	Tjp1	1	15
NM_008481	Laminin subunit alpha	Lama 2	2	43
NM_016701	Nestin	Nes	1	13
NM_016721	Ras GTPase-activating-like protein	IQGAP1	1	12
NP_001343322	Clathrin heavy chain 1	Cltc	2	40
XM_006516009	HEAT repeat-containing protein 5a	Heatr 5a	1	15
NM_178704	Dpy-19-like 3	Dpy19l3	2	33

Supplementary Table 2. List of oligo primers used in this study

Primer name	Sequence(5'→3')
Pgk1 F	ATGTCGCTTTCCAACAAGCTGACTTTGGAC
Pgk1 R	CTAAACATTGCTGAGAGCATCCACCCCAGG
zPgk1 F	ATGTCTCTTTCGAACAAACTTCATTTGGAC
zPgk1 R	TTATACGTTACTGAGGGCATCGACACCGGG
Eno2 F	GAATTCATGTCTATAGAGAAGATTTGGGCCCGAGAGATCTT
Eno2 R	CGGGACATAATTTCCGAAATCCCAGTGTGCTGTGACTCGAG
TRPC5 F	ATGGCCCAGCTGTACTACAAGAAGGTCAAT
TRPC5 R	TTAGAGCCGAGTTGTAACTTGTTCTTCCTG
Tlr9 F	ATGGTTCTCCGTCGAAGGACTCTGCACCCC
Tlr9 R	CTATTCTGCTGTAGGTCCCCGGCAGAAGTT
eno2 F	ATATAATCGATATGTCTGTTGTAAGCATCATTGCCAGGG
eno2-Myc R	ATATAGAATTCTCACAGATCCTCTTCAGAGATGAGTTTCTGCTCCAG AGCGCTGGGGTTTCTGAAGTTG
eno3 F	ATATAGAATTCATGTCCATTAGTAAGATTCACGCTCGTGAGATC
eno3-Myc R	ATATACTCGAGCTACTTATCGTCGTCATCCTTGTAATCGAGTTTGGG GTGGCGGAAGTCTTTTC
Pgk1(1-145) R	GGCCGGCTCAGCTTTAACCTTGTTTCCAGAAGCAT
Pgk1(146-417) F	ATGAAAATTGATGCTTTCCGAGCCTCACTGTCCAAACT
Pgk1(225-417) F	ATGAATAATATGCTAGACAAAGTCAATGAGATGATCAT
Pgk1(325-417) F	ATGGCCGAGGCTGTGGGTCGAGCTAAGCAGATTGTTTG
Eno2△ F	ATATAGGATCCATGTCTATAGAGAAGATTTGGGCCCGAG
Eno2△405-431-Myc R	ATATAGAATTCTCACAGATCCTCTTCAGAGATGAGTTTCTGCTCCAG CACACTACGTTCAGATCTGCATGGGGCG
Eno2△432-434-Myc R	ATATAGAATTCTCACAGATCCTCTTCAGAGATGAGTTTCTGCTCGGG ATTTCGGAAATTATGTCCCGCG
eno2△ F	ATATAATCGATATGTCTGTTGTAAGCATCATTGCCAGGG
eno2△405-431-Myc R	ATATAGAATTCTCACAGATCCTCTTCAGAGATGAGTTTCTGCTCCAG AGCGCTACGCTCAGATCTACATGGAGCT
eno2△432-434-Myc R	ATATAGAATTCTCACAGATCCTCTTCAGAGATGAGTTTCTGCTCGGG GTTTCTGAAGTTGTGCCCGG
Eno1-EcoRI-F	TATATGAATTCATGTCTATTCTCAGGATCCACGCCAG3
Eno1-myc-NotI-R	TATATGCGGCCGCTTACAGATCCTCTTCTGAGATGAGTTTTTGTTCTT TGGCCAGGGGGTTCCTGAAG
