# Supplementary material for: Extracellular Pgk1 interacts neural membrane protein enolase-2 to improve the neurite outgrowth of motor neurons
Source: Commun Biol. 2023 Aug 15;6:849. doi: 10.1038/s42003-023-05223-0 (PMC10427645; doi:10.1038/s42003-023-05223-0)
Supplement: Supplementary file 5 — Reporting summary [file 42003_2023_5223_MOESM5_ESM.pdf]

nature portfolio

Corresponding author(s): COMMSBIO-22-4086B Huai-Jen Tsai

Last updated by author(s): Jul 28, 2023

Reporting Summary

Nature Portfolio wishes to improve the reproducibility of the work that we publish. This form provides structure for consistency and transparency in reporting. For further information on Nature Portfolio policies, see our Editorial Policies and the Editorial Policy Checklist.

Statistics

For all statistical analyses, confirm that the following items are present in the figure legend, table legend, main text, or Methods section.

n/a	Confirmed	
☐	☒	The exact sample size (*n*) for each experimental group/condition, given as a discrete number and unit of measurement
☐	☒	A statement on whether measurements were taken from distinct samples or whether the same sample was measured repeatedly
☐	☒	The statistical test(s) used AND whether they are one- or two-sided<br>*Only common tests should be described solely by name; describe more complex techniques in the Methods section.*
☒	☐	A description of all covariates tested
☒	☐	A description of any assumptions or corrections, such as tests of normality and adjustment for multiple comparisons
☐	☒	A full description of the statistical parameters including central tendency (e.g. means) or other basic estimates (e.g. regression coefficient) AND variation (e.g. standard deviation) or associated estimates of uncertainty (e.g. confidence intervals)
☐	☒	For null hypothesis testing, the test statistic (e.g. *F*, *t*, *r*) with confidence intervals, effect sizes, degrees of freedom and *P* value noted<br>*Give P values as exact values whenever suitable.*
☒	☐	For Bayesian analysis, information on the choice of priors and Markov chain Monte Carlo settings
☒	☐	For hierarchical and complex designs, identification of the appropriate level for tests and full reporting of outcomes
☒	☐	Estimates of effect sizes (e.g. Cohen's *d*, Pearson's *r*), indicating how they were calculated

Our web collection on statistics for biologists contains articles on many of the points above.

Software and code

Policy information about availability of computer code

Data collection	software used for data collection is cited in the manuscript and supplementary information
Data analysis	ImageJ, MetaMorph, TMHMM-2.0, ProtScale-ExPASy, Microsoft Excel

For manuscripts utilizing custom algorithms or software that are central to the research but not yet described in published literature, software must be made available to editors and reviewers. We strongly encourage code deposition in a community repository (e.g. GitHub). See the Nature Portfolio guidelines for submitting code & software for further information.

Data

Policy information about availability of data

All manuscripts must include a data availability statement. This statement should provide the following information, where applicable:

- Accession codes, unique identifiers, or web links for publicly available datasets
- A description of any restrictions on data availability
- For clinical datasets or third party data, please ensure that the statement adheres to our policy

The authors declare that the data supporting the findings of this study are available within the paper and its supplementary information files.

Human research participants

Policy information about studies involving human research participants and Sex and Gender in Research.

Reporting on sex and gender	NOT APPLICABLE
Population characteristics	NOT APPLICABLE
Recruitment	NOT APPLICABLE
Ethics oversight	NOT APPLICABLE

Note that full information on the approval of the study protocol must also be provided in the manuscript.

Field-specific reporting

Please select the one below that is the best fit for your research. If you are not sure, read the appropriate sections before making your selection.

☒ Life sciences ☐ Behavioural & social sciences ☐ Ecological, evolutionary & environmental sciences

For a reference copy of the document with all sections, see nature.com/documents/nr-reporting-summary-flat.pdf

Life sciences study design

All studies must disclose on these points even when the disclosure is negative.

Sample size	All experiments were reproduced at least three times with similar results, The sample size and how many times each experiment was performed are indicated in the Figure legends. Each our experiments
Data exclusions	No data were excluded from the analyses
Replication	All experiments were reproduced at least three times with similar results, The sample size and how many times each experiment was performed are indicated in the Figure legends. Each our experiments
Randomization	zebrafish embryos were randomly assigned to groups for each experiment.
Blinding	Blinding was not performed prior to experimentation

Reporting for specific materials, systems and methods

We require information from authors about some types of materials, experimental systems and methods used in many studies. Here, indicate whether each material, system or method listed is relevant to your study. If you are not sure if a list item applies to your research, read the appropriate section before selecting a response.

Materials & experimental systems

n/a	Involved in the study	
☐	☒	Antibodies
☐	☒	Eukaryotic cell lines
☒	☐	Palaeontology and archaeology
☐	☒	Animals and other organisms
☒	☐	Clinical data
☒	☐	Dual use research of concern

Methods

n/a	Involved in the study	
☒	☐	ChIP-seq
☒	☐	Flow cytometry
☒	☐	MRI-based neuroimaging

Antibodies

Antibodies used	Antibodies against phosphorylated Cofilin at S3 (RRID:AB_2080597; 1:1000), Cofilin (RRID:AB_10622000; 1:1000), phosphorylated Limk1 at S323 (RRID:AB_2136940; 1:1000), phosphorylated Limk1 at T508 (RRID:AB_2136943; 1:500), LIM domain kinase 1 (Limk1) (RRID:AB_648350; 1:500), phosphorylated p38 at T180 (RRID:AB_331641; 1:1000), p38 mitogen-activated protein kinases (p38) (RRID:AB_330713; 1:1000), Eno1 (RRID:AB_1118874; 1:1000), Eno2 (NSE-P1) (RRID:AB_627513; 1:1000), eno2 (SC06-28; 1:1000) , goat anti-mouse-HRP (RRID:AB_955439; 1:5000), goat anti-rabbit-HRP (RRID:AB_631746; 1:5000) and chicken anti-goat-HRP (RRID: AB_639230; 1:5000) were used. Antibody against α-tubulin (RRID:AB_477579; 1:1000) served as an internal control.

Validation	Data on validation of deplenting antibodies is provided in the manuscipt

Eukaryotic cell lines

Policy information about cell lines and Sex and Gender in Research

Cell line source(s)	We thank Dr. Hsi-Yuan Yang, University of Taiwan, for providing HEK293T cell line.<br>We thank Dr. Neil Cashman, University of Toronto, for providing NSC34 cell line.<br>We thank Dr. Yen-Ling Song, University of Taiwan, for providing Sf21 cell line.
Authentication	cell lines were not authenticated
Mycoplasma contamination	cells tested negative for mycoplasma
Commonly misidentified lines<br>(See ICLAC register)	Commonly misidentified lines were not used in this study

Animals and other research organisms

Policy information about studies involving animals; ARRIVE guidelines recommended for reporting animal research, and Sex and Gender in Research

Laboratory animals	Tg(mnx:GFP) zebrafish (Danio rerio) were used in this study.
Wild animals	NOT APPLICABLE
Reporting on sex	NOT APPLICABLE
Field-collected samples	NOT APPLICABLE
Ethics oversight	The experiments and treatments of this zebrafish model have been reviewed and approved by the Fu Jen Catholic University and Mackay Medical College Institutional Animal Care and Use Committee with ethics approval number A11064 and A1050012, respectively.

Note that full information on the approval of the study protocol must also be provided in the manuscript.
